# The threat of reduced efficacy of anthelmintics against gastrointestinal nematodes in sheep from an area considered anthelmintic resistance-free

**DOI:** 10.1186/s13071-020-04329-2

**Published:** 2020-09-09

**Authors:** Antonio Bosco, Jan Kießler, Alessandra Amadesi, Marian Varady, Barbara Hinney, Davide Ianniello, Maria Paola Maurelli, Giuseppe Cringoli, Laura Rinaldi

**Affiliations:** 1 Department of Veterinary Medicine and Animal Production, University of Naples Federico II, CREMOPAR, Naples, Italy; 2 Institute of Parasitology, Department of Pathobiology, University of Veterinary Medicine Vienna, 1210 Vienna, Austria; 3 Institute of Parasitology of Slovak Academy of Sciences, 040 01 Košice, Slovakia

**Keywords:** Sheep, Gastrointestinal nematodes, Anthelmintic resistance, Pooling faecal samples, Faecal egg count reduction test, Egg hatch test

## Abstract

**Background:**

The worldwide increased difficulty to combat gastrointestinal nematode (GIN) infection in sheep, due to progressing anthelmintic resistance (AR), calls for an enhanced and standardized implementation of early detection of AR. This study provides a snapshot of the current AR status against benzimidazoles and macrocyclic lactones in southern Italy, generated with standardized techniques.

**Methods:**

On 10 sheep farms, the efficacy of albendazole (ALB) and either eprinomectin (EPR) or ivermectin (IVM) was evaluated based on the faecal egg count reduction test (FECRT) performed with the Mini-FLOTAC. For each tested drug, 40 sheep were rectally sampled at D0 and sampled again 14 days after the treatment (D14). The FECRT was calculated from individual samples and pooled samples which consist of 5 individual samples. Efficacy was classified as ‘reduced, ‘suspected’ and ‘normal’. Coprocultures were set for D0 and D14 faecal samples of each group. From farms with FECR < 95%, an *in vitro* egg hatch test (EHT) and a follow-up FECRT using fenbendazole (FBZ) were conducted.

**Results:**

Based on the FECR, high efficacy (from 95.7% to 100%) was observed for ALB and IVM in eight farms (Farms 3–10). On Farm 1 and Farm 2, the efficacy for the macrocyclic lactones was classified as ‘normal’, but ‘reduced’ efficacy was observed for ALB on Farm 1 (FECR = 75%) and ‘suspected’ efficacy on Farm 2 (FECR = 93.3%) with the predominant GIN genus *Trichostrongylus* followed by *Haemonchus* at D14. The FEC results of pooled samples strongly correlated with those of individual samples, for FEC at D0 (*r*_*s*_ = 0.984; *P* < 0.0001) and at D14 (*rs* = 0.913; *P* < 0.0001). The classifications of efficacy in Farm 1 (FECR = 86.0%) and Farm 2 (FECR = 93.0%) in the follow-up FECRT with FBZ coincide with the main FECRT trial. The *in vitro* EHT confirmed AR in both farms (Farm 1: 89%; Farm 2: 74%).

**Conclusions:**

In regions like southern Italy, where the negative impacts from AR have played a minor role, efficient monitoring of AR is important in order to evaluate potential risks and being able to promptly respond with countermeasures.
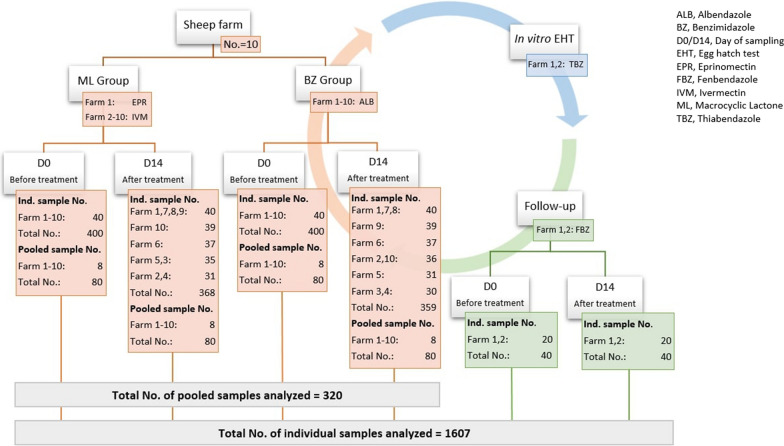

## Background

Gastrointestinal nematode (GIN) infection endangers livestock health and welfare and is commonly associated with economic losses mostly through subclinical diseases impairing weight gain and milk yields [1–4]. GIN parasites are considered endemic and have no major regulatory or trade implications, thus their control has largely remained the responsibility of the farmers and/or the veterinarians [4].

Management and control of GIN infections is a challenging task and currently dependent almost only on efficient anthelmintic drugs. However, the improper use (over- and mis-use) of anthelmintics has led to development of anthelmintic resistance (AR) which is now reported worldwide in multiple nematode species, especially in sheep, against multiple anthelmintic classes, e.g. benzimidazoles (BZ) and macrocyclic lactones (ML) ([3, 5–8], Hanna Rose Vineer et al. in preparation).

Due to the costs of anthelmintic-resistant nematode infections, there is a wide consensus on the need to enhance and implement early detection of AR based on active monitoring of the efficacy of anthelmintics in order to promptly respond to the development of AR. There are indications that some actions are able to slow down the development and spread of AR, e.g. promote “best practice” parasite management programmes based on sustainable use of anthelmintics through targeted treatment (TT) and targeted selective treatment (TST) based on easy-to-use diagnostics to inform treatment decisions [7, 9].

In Italy, few reports of AR in sheep against levamisole, ivermectin and benzimidazoles have been published but mainly in northern and central regions [10–12]. On the contrary, in southern Italy some concrete actions appear to be effective in maintaining the efficacy of anthelmintics and slowing the development of AR, e.g. the monitoring of GIN infection in sheep and other livestock by regular diagnosis, use of targeted treatments, rotation of different drugs, correct drenching, and low movement of animals between farms [13–15]. However, given that the development of AR is inevitable and its occurrence is not a matter of “if” but “when” (R. Kaplan, personal communication), the aim of this study was to investigate the current levels of efficacy of BZ and ML in sheep in the Campania region (southern Italy) by performing a standardized survey by the faecal egg count reduction test (FECRT) in accordance with the guidelines established in the framework of the European COST Action “COMBAR - COMBatting Anthelmintic Resistance in Ruminants” (https://www.combar-ca.eu).

In particular, the FECRT was performed by the Mini-FLOTAC technique [16] on individual and pooled samples [17, 18]. Mini-FLOTAC is an easy-to-use yet sensitive and accurate diagnostic method for FECRT which, complemented with *in vitro* and molecular tools, is able to give a precise measure of AR [19]. In the farms where BZ resistance was suspected (i.e. reduced efficacy of the drug), confirmation was done by *in vitro* tests and a follow-up *in vivo* trial.

In doing so, the study met current recommendations in the experimental setup [20] and made a step towards a standardized approach in evaluating anthelmintic efficacy and AR in grazing ruminants.

## Methods

### Study area

The study was conducted in the Campania region of southern Italy where the climate is characterized by dry summers and rainy autumns/winters. The area is mainly used for cereal production but small pastures occur on upland areas that are unsuitable for cropping. Small ruminant production systems are a major component of the dairy and meat sector in the region and each sheep farm is approximately 50 ha.

### Study farms and animals

Trials were conducted between July and October 2019 on 10 sheep farms. Dairy sheep farms were selected throughout the region and the selection was mainly driven by the availability of the farmers and the presence of GIN positive sheep. The average flock size of the selected ten farms was of 250 sheep (range 100–700 animals). All farmers bred the sheep for milk production. Three flocks were composed of Lacaune mixed-breed dairy sheep, five of Bagnolese mixed-breed dairy sheep and two Lacaune/Bagnolese/Sarda/Comisana mixed-breed dairy sheep. Animals of all farms had access to pasture for the entire year. All farmers conducted whole-flock anthelmintic treatments twice per year, first during the dry-off period i.e. in the peripartum period (October/November or February/March) and second in May/June.

In each farm the animals were divided into 2 groups of 40 sheep randomly chosen, one group treated with ML (ivermectin/eprinomectin) and one group with BZ (albendazole), without using an untreated control group [21]. On each farm the enrolled animals were individually weighed and the correct dose of drugs was administered using an appropriate equipment, calibrated to deliver the dose accurately. Specifically, in Farm 1, 40 sheep were treated with an oral suspension of albendazole (ALB, Valbazen^®^ Zoetis, Rome, Italy; 3.8 mg/kg of body weight) and 40 with a pour on solution of eprinomectin (EPR, Eprinex Multi^®^ Boehringer Ingelheim Animal Health, Milan, Italy; 1.0 mg/kg of body weight). In the other 9 farms 40 sheep were treated with ALB (ALB, Valbazen^®^ Zoetis; 3.8 mg/kg of body weight) and 40 with an oral solution of ivermectin (IVM, Oramec^®^ Boehringer Ingelheim Animal Health; 0.2 mg/kg of body weight).

**Fig. 1:**
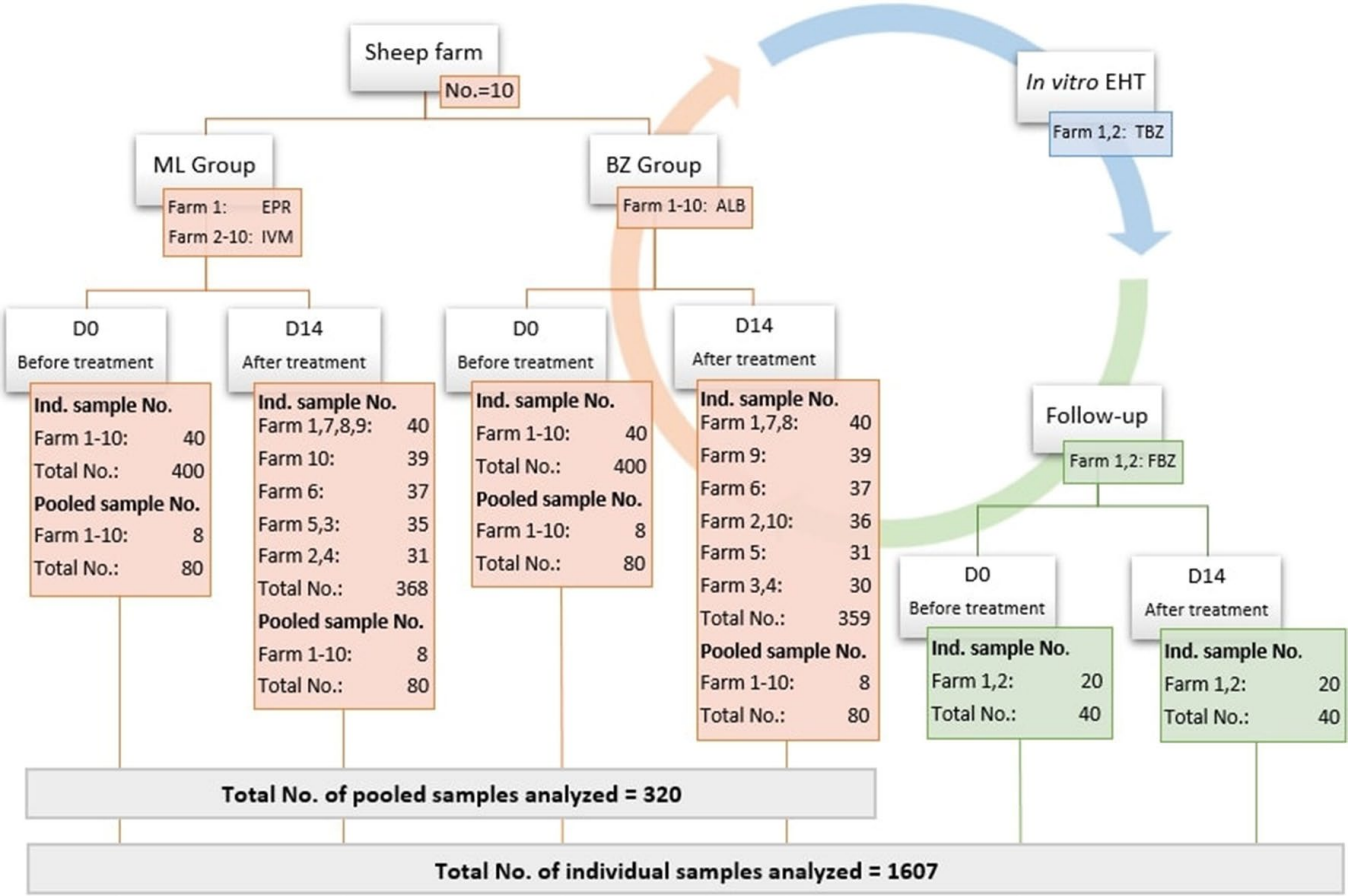
The number of sheep farms, anthelmintics used, sampling time and number of samples per farm used for the field trial setup, follow-up trial and in *vitro* egg hatch tests

Individual faecal samples were collected rectally on the day of treatment (D0) and after 14 days (D14), stored shortly thereafter at 4 °C and further laboratory processed as individual and pooled samples as soon as possible. The number of farms, individual and pooled faecal samples used in this study are provided in Fig. 1.

### Laboratory procedures

At D0 and D14, the ovine faecal samples were analysed both individually and as pooled samples using the Mini-FLOTAC technique with a detection limit of 5 eggs per gram (EPG) of faeces, using a sodium chloride flotation solution (FS2, specific gravity = 1.200) [16]. The pool size consisted of 5 individual samples according to the protocol described in Rinaldi et al. [17]. Each sample was labelled, thoroughly homogenized, individually examined and then composite (pooled) samples were prepared taking 5 g of each sample with the collector of the Fill-FLOTAC. It should be noted that the predefined pool sizes of 5 could not always be met at D14 due to insufficient amount of faeces to perform the analysis of each pool.

### FECRT

When examining individual samples and pooled samples, the FECR (%) and 95% CI were calculated using the “eggCounts-2.3” on R version 3.6.1. [22] considering individual FECs before and after treatment (two paired samples) for each group, correction factor of 5 (Mini-FLOTAC analytical sensitivity) and no zero inflation.

Drug efficacy was classified as ‘reduced’ when FECR < 95% and the lower limit (LL) of the 95% confidence interval (CI) < 90%, as ‘suspected’ when either FECR < 95% or LL < 90% and as ‘normal’ when FECR ≥ 95% and LL ≥ 90% [21, 23].

### Coprocultures

Before the storage at 4 °C, the same quantity of faeces was collected from each sample to create a pool for each faecal culture group at D0 and D14, following the protocol described by the Ministry of Agriculture, Fisheries and Food [24]. Developed third-stage larvae (L3) were identified using the morphological keys proposed by van Wyk & Mayhew [25]. Identification and percentages of each nematode genera were conducted on 100 L3; if a sample had 100 or less L3 present, all larvae were identified. Thus, on the total number of larvae identified, it was possible to give the percentage of each genus.

### Follow-up study

The farms, on which benzimidazole efficacy has been classified as ‘reduced’ (Farm 1) and as ‘suspected’ (Farm 2) through the *in vivo* trial (see the Results section), were revisited after a period of 2 months. Faecal samples were sampled as a follow-up to the *in vivo* study. The follow-up samples were taken from 20 individual sheep randomly chosen from each farm. The sheep were treated with a drug of the same anthelmintic class (side resistance) that showed low efficacy (fenbendazole-FBZ was used instead of ALB). Fourteen days after drug application, the same group of 20 animals were sampled a second time by collecting the faeces from the rectum. All samples were handled as described previously in the laboratory procedures.

### Egg hatch test

To confirm the results of the FECRT an *in vitro* assay, i.e. the egg hatch test (EHT), was performed in Farm 1 and Farm 2 where BZ resistance was suspected (see the Results section).

GIN eggs were recovered from follow-up samples collected directly from the rectal ampulla of 30–40 sheep on each farm. The faecal samples were processed within 2 h of collection by using the egg recovery technique as described by Coles et al. [23] with some modifications.

Firstly, faecal samples were homogenized and filtered under running water through sieves with a mesh size of 125, 63 and 38 µm (CISA Sieving Technologies, Barcelona, Spain) in order to separate the eggs from the faeces. Next, the GIN eggs retained on the last sieve were washed and centrifuged for 3 min at 170×*g* with distilled water, after which the supernatant was discarded. In the end, centrifugation was performed using 40% sugar solution to float the eggs which are then isolated in new tubes, mixed with distilled water and then centrifuged two more times in order to remove pellets and to get aqueous solution with eggs.

Eggs were inspected microscopically to record if embryonation had not begun. Each sample was at least tested in duplicate and at least two negative control samples were used (eggs with DMSO). A stock solution of thiabendazole (TBZ) was prepared by dissolving the pure compound in dimethyl sulfoxide (DMSO) and following the protocol described by [26].

The final concentrations in the EHT were prepared by adding 10 µl of each TBZ solution into 1.99 ml of a suspension with approximately 150 eggs/ml in water. The final TBZ concentrations used were 0.5, 0.3, 0.2, 0.1, 0.05, 0.025 and 0.01 μg/ml. A control (0.5% DMSO) without anthelmintic was also included in the test. The 24-well tissue culture test plates (Corning Incorporated, Life sciences, Salt Lake City, USA) were incubated for 48 h at 25 °C. The incubation was then terminated by adding 10 µl of Lugol’s iodine solution to each well. After 48 h, at least 100 eggs (dead, embryonated) and hatched first-stage larvae in each well were counted. The test was performed with two replicates.

### Statistical analysis

The statistical analysis was performed with the *egg-Counts*-package in R [22]. The paired model was used to calculate FECR and 95% confidence intervals (95% CI) using individual FECs before and after treatments for each single group.

The arithmetic mean FEC of individual and pooled samples were calculated. Correlations between the different measures of FEC were assessed by Spearman’s rho correlation coefficient (*r*_*s*_), the associated 95% CI and *P*-value. Moreover, Lin’s concordance correlation coefficients (CCC) and the corresponding 95% CI were calculated to quantify the agreement between the analysis from the FEC of individual samples and the FEC of pooled samples. Spearman’s *r*_*s*_ and Lin’s CCC were calculated as above between FECR (%) from individual and pooled samples. Like a correlation, CCC ranges from -1 to 1, with perfect agreement at 1. The strength of agreement was classified as poor, moderate, substantial or almost perfect for CCC values < 0.9, 0.90–0.95, 0.95–0.99, and > 0.99, respectively [27].

A four-parameter logistic equation with a variable slope was chosen for the statistical analysis of *in vitro* test results. All analyses were performed after transforming the TBZ concentrations into its logarithm (X = logX) and defining the bottom value 0. The EHT protocol recommended as method for the detection of BZ resistance with cut off of > 0.1 µg TBZ per ml for 50% egg hatch inhibition (EC_50_) [23].

## Results

### History of parasite management on farms

Only two farms (Farm 1 and Farm 2), having had a known history of fasciolosis [28], used a strategic schemes of anthelmintic treatments based only on the use of ALB (four times a year in February/March, May/June, August/September and November/December) over six years to reduce the prevalence and intensity of *Fasciola hepatica* to a level, at which there were no longer any clinical symptoms of the disease.

Seven farmers used to practice regular parasitological monitoring before the anthelmintic treatment and all performed a rotation of anthelmintics using benzimidazoles and macrocyclic lactones once per year; one class was used in spring, the other class in autumn and *vice versa*. Only four farmers used coprological analysis for efficacy control.

### FECRT

**Fig. 2:**
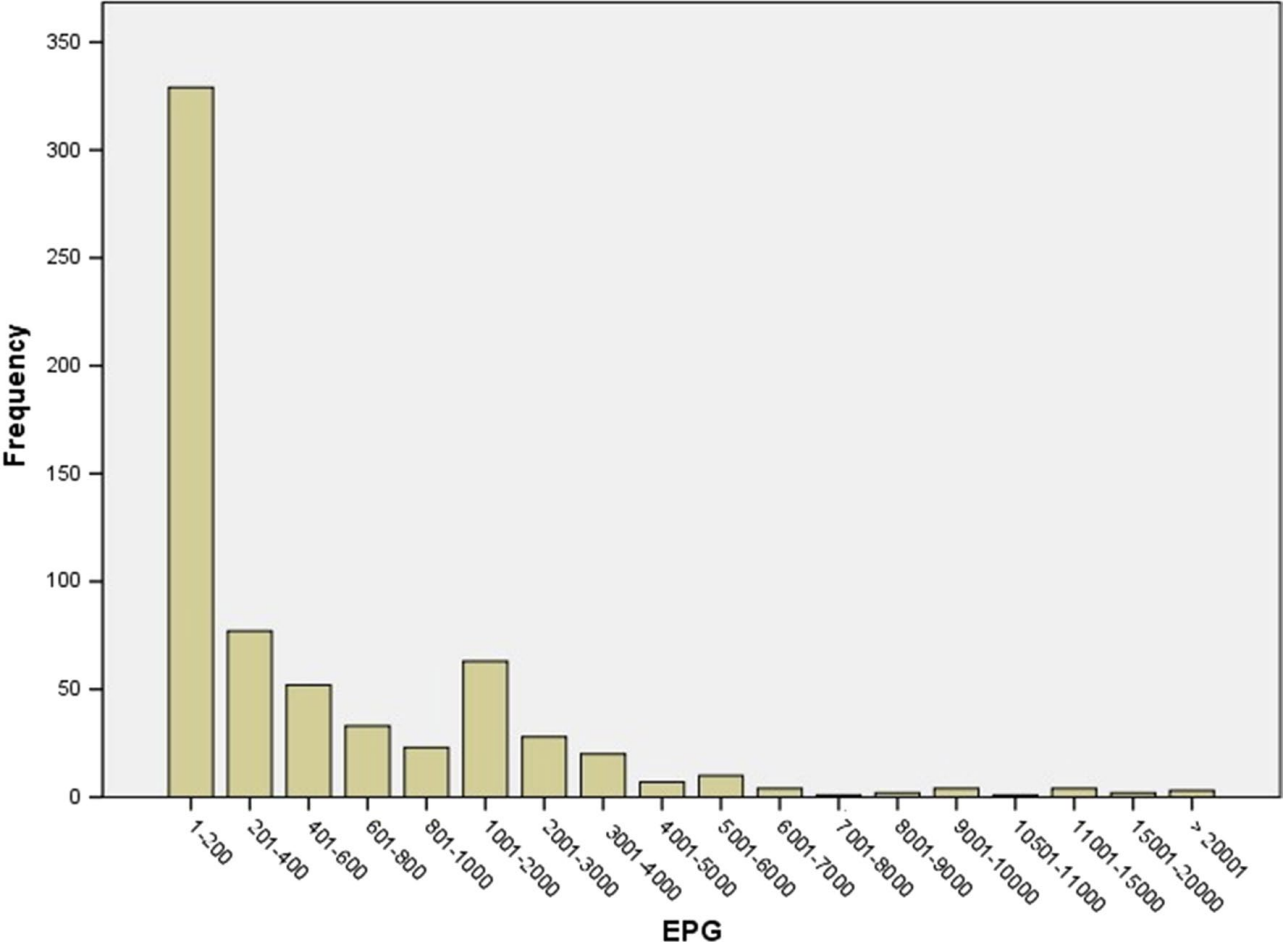
Frequency distribution of gastrointestinal nematode (GIN) egg counts among different individual animals of all examined farms sampled at D0

**Fig. 3:**
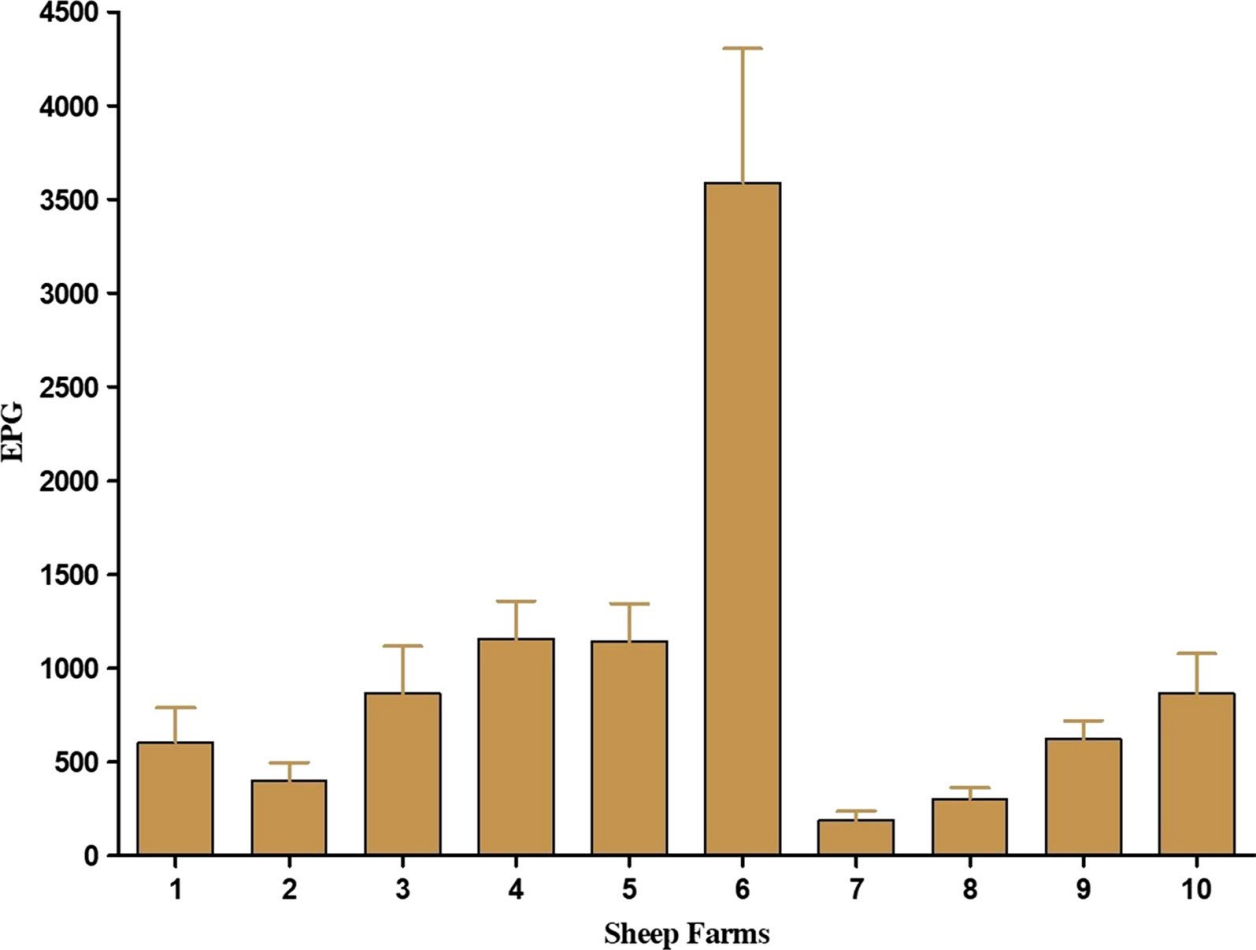
Variability of gastrointestinal nematode (GIN) egg counts in the different sheep farms sampled at D0: mean eggs per gram (EPG) of faeces and standard errors (SE)

The frequency of FEC distribution of individual samples at D0, described in Fig. 2, showed that 70% of animals are responsible for shedding GIN eggs from 1 to 600 EPG while 30% of the animals are responsible for shedding most of GIN eggs (from 601 to 35,520 EPG). Furthermore, the mean EPG values and the standard error for each farm involved in this study, described in Fig. 3, showed a great variability among the different farms used in this study, with a mean of GIN EPG per farm from 188 to 3590.

The efficacies of the anthelmintic treatments are given in Table 1. Very high efficacy was obtained with the two classes of anthelmintics tested in 8 farms as follows (farm average FECR, min and max): IVM 99.1% (95.7–100%) and ALB 99.4% (97.4–100%). On all 8 farms lower confidence limits (LL) were generally high and always above 95.1% for IVM and 98.0% for ALB.

**Table 1:** The anthelmintics (molecules and dosages) used on each group of 10 sheep farms against gastrointestinal nematodes (GIN), mean GIN eggs per gram (EPG) of faeces (day 0 and day 14), results of the faecal egg count reduction (FECR%), limits of the 95% confidence interval (CI) of individual and pooled faecal samples and anthelmintic efficacy classified as reduced (R), suspected (S) and normal (N)

Farm ID	Group	Molecule	Dosage (mg/kg)	Individual samples				Pooled samples				Efficacy
				D0 FEC Mean EPG	D14 FEC Mean EPG	FECR %	95% CI	D0 FEC Mean EPG	D14 FEC Mean EPG	FECR %	95% CI	
1	EPR	Eprinomectin	1.0	1086	25	97.7	97.4–98.0	1100	23	97.9	97.1–98.5	N
	ALB	Albendazole	3.8	124	31	75.0	71.2–78.2	116	30	73.7	64.3–81.0	R
2	IVM	Ivermectin	0.2	417	0.5	99.9	99.7–100	380	0	99.9	99.4–100	N
	ALB	Albendazole	3.8	375	25	93.3	93.0–94.8	344	25	92.6	89.9–94.7	S
3	IVM	Ivermectin	0.2	540	3	99.4	99.2–99.6	478	3	99.1	98.3–99.6	N
	ALB	Albendazole	3.8	1195	31	97.4	99.5–99.6	1189	35	97.0	96.2–97.7	N
4	IVM	Ivermectin	0.2	965	1	99.9	99.7–99.9	885	1	99.7	99.3–99.9	N
	ALB	Albendazole	3.8	1348	0	100	99.9–100.0	1116	0	100	99.6–100	N
5	IVM	Ivermectin	0.2	1863	42	97.7	97.3–97.9	1816	40	97.8	97.2–98.3	N
	ALB	Albendazole	3.8	420	1	99.8	99.6–99.9	401	0	99.6	98.9–99.9	N
6	IVM	Ivermectin	0.2	3437	28	99.2	99.1–99.3	3609	40	97.8	97.4–98.2	N
	ALB	Albendazole	3.8	3742	16	99.6	99.5–99.6	3799	13	99.6	99.5–99.8	N
7	IVM	Ivermectin	0.2	102	0	100	99.9–100	88	0	99.5	97.3–100	N
	ALB	Albendazole	3.8	274	0	100	99.9–100	202	0	99.8	98.8–100	N
8	IVM	Ivermectin	0.2	272	0	100	99.9–100	272	0	99.8	99.2–100	N
	ALB	Albendazole	3.8	334	0	100	99.9–100	334	0	100	99.3–100	N
9	IVM	Ivermectin	0.2	454	0	100	99.9–100	434	0	100	99.5–100	N
	ALB	Albendazole	3.8	796	13	98.4	98.0–98.6	636	14	97.7	96.6–98.5	N
10	IVM	Ivermectin	0.2	1059	46	95.7	95.1–96.3	1023	33	96.7	95.8–97.5	N
	ALB	Albendazole	3.8	669	0	100	99.9–100	605	0	100	99.6–100	N

In Farm 1 and Farm 2 two macrocyclic lactones used (EPR and IVM) showed an efficacy of 97.9% and 99.9%, respectively. While on both farms a low efficacy of ALB was observed (in Farm 1 of 75.0% with LL of 71.2% and in Farm 2 of 93.3% with LL of 93.0%) and AR was present in Farm 1 (reduced) and suspected in Farm 2. In addition, the follow-up trial using FBZ also showed low efficacy of benzimidazoles in both farms (Table 2).

**Table 2:** Fenbendazole dosage, mean gastrointestinal nematode (GIN) eggs per gram (EPG) of faeces (day 0 and day 14), results of the faecal egg count reduction (FECR, %), limits of the 95% confidence interval (CI) and anthelmintic efficacy classified as reduced (R), suspected (S) and normal (N) in the follow-up study

Farm ID	Group	Molecule	Dosage (mg/kg)	D0 FEC (Mean EPG)	D14 FEC (Mean EPG)	FECR%	95% CI	Efficacy
1	FBZ	Fenbendazole	5.0	194	27	86.0	82.9–88.6	R
2	FBZ	Fenbendazole	5.0	151	11	93.0	90.4–94.9	S

**Fig. 4:**
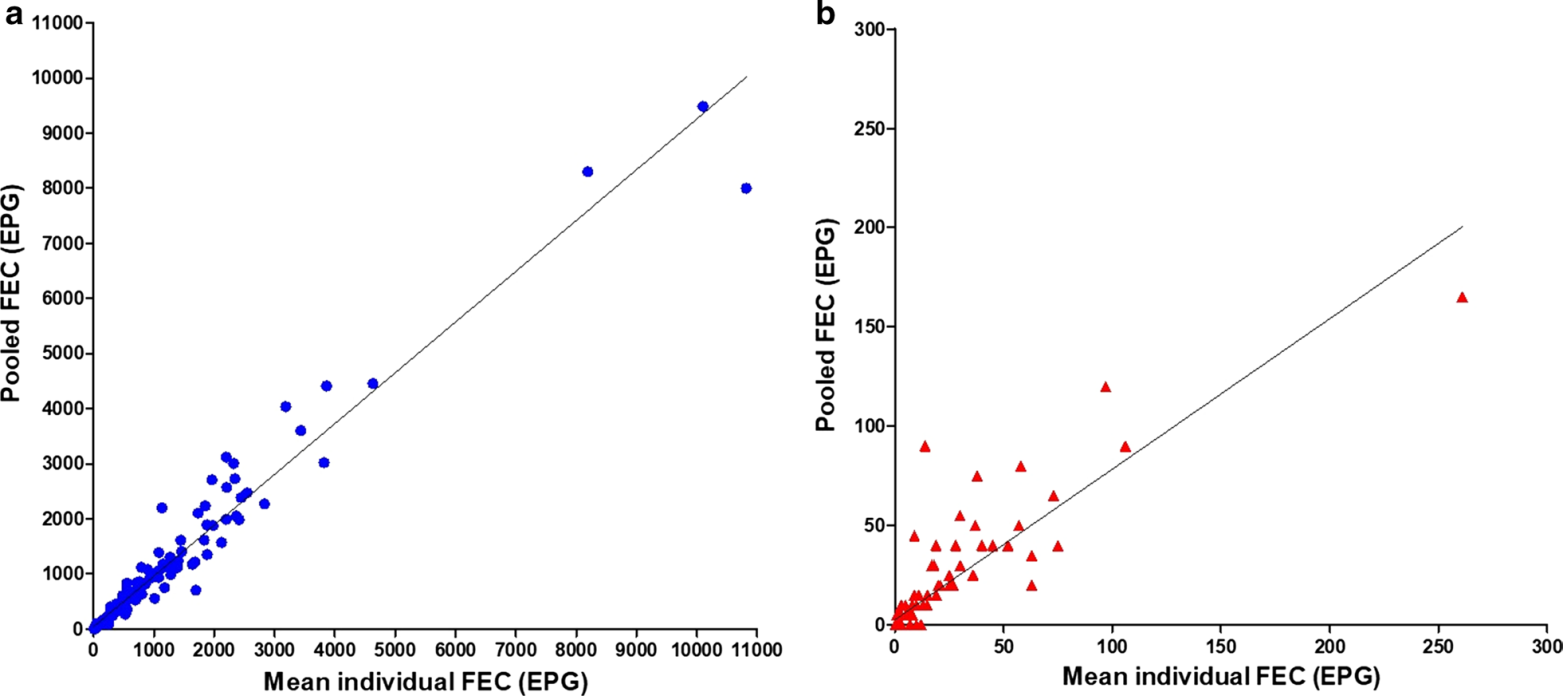
The correlation in faecal egg counts at D0 (**a**) and at D14 (**b**) based on the examination of individual and pooled faecal samples of all examined farms

When calculated from individual samples, the GIN FEC mean values at D0 ranged from 7 to 10,819 EPG and at D14 ranged from 0 to 261 EPG, whilst when calculated from pooled samples the GIN FEC mean values at D0 ranged from 5 to 9485 EPG and at D14 ranged from 0 to 165 EPG. The correlation between FEC results from individual means and pool means is reported in Fig. 4. When considering results separately for D0 or D14, the FEC results of pooled samples strongly correlated with those of individual samples, for FEC at D0 (*r*_*s*_ = 0.984, 95% CI: 0.978–0.987, *P* < 0.0001) and at D14 (*r*_*s*_ = 0.913, 95% CI: 0.879–0.937, *P* < 0.0001). The level of agreement between the FEC from individual and pool means was substantial for FEC at D0 (CCC = 0.973, 95% CI: 0.963–0.980) and poor for FEC at D14 (CCC = 0.873, 95% CI: 0.830–0.905). Overall, when considering results including FEC at D0 and FEC at D14 results showed a high correlation (*r*_*s*_ = 0.987, 95% CI: 0.984–0.990, *P* < 0.0001) and a substantial level of agreement (CCC = 0.978, 95% CI: 0.972–0.982, *P* < 0.001).

The FECR (%) considering individual samples ranged between 75 and 100%, whilst considering pooled samples ranged between 73.7 and 100% and the correlation between FECRs resulting from individual and pooled samples showed an high *r*_*s*_ value (*r*_*s*_ = 0.924, 95% CI: 0.809–0.976, *P* < 0.0001) and a substantial CCC value (CCC = 0.995, 95% CI: 0.989–0.998).

### Coprocultures

The genera of nematodes present (minimum and maximum percentages in each treatment group) at D0 were *Haemonchus* (21–83%), *Trichostrongylus* (2–59%), *Teladorsagia* (0–25%), *Chabertia* (0–48%) and *Cooperia* (0–5%), whilst very few numbers of nematode L3 were found at D14 on groups of other farms (Table 3) at D14. In the two farms where AR for ALB was suspected, the following GIN genera were detected: *Haemonchus* (7–29%) and *Trichostrongylus* (93–71%).

**Table 3:** Numbers and percentage of sheep nematode third-stage larvae (L3) for each group of 10 sheep farms at D0 and at D14

Farm ID	Group	Day	*Haemonchus*		*Trichostrongylus*		*Teladorsagia*		*Cooperia*		*Chabertia*	
			*n*	%	*n*	%	*n*	%	*n*	%	*n*	%
1	EPR	0	78	78	19	19	1	1	0	0	2	2
		14	7	7	93	93	0	0	0	0	0	0
	ALB	0	79	79	21	21	0	0	0	0	0	0
		14	29	29	71	71	0	0	0	0	0	0
2	IVM	0	77	77	20	20	1	1	0	0	2	2
		14	6	24	19	76	0	0	0	0	0	0
	ALB	0	78	78	17	17	2	2	0	0	3	3
		14	6	6	94	94	0	0	0	0	0	0
3	IVM	0	67	67	15	15	18	18	0	0	0	0
		14	10	50	7	35	3	15	0	0	0	0
	ALB	0	72	72	8	8	20	20	0	0	0	0
		14	59	59	29	29	7	7	0	0	5	5
4	IVM	0	67	67	23	23	6	6	0	0	4	4
		14	0	0	11	61	7	39	0	0	0	0
	ALB	0	68	68	15	15	8	8	0	0	9	9
		14	9	82	2	18	0	0	0	0	0	0
5	IVM	0	73	73	15	15	12	12	0	0	0	0
		14	73	73	19	19	8	8	0	0	0	0
	ALB	0	21	21	54	54	25	25	0	0	0	0
		14	11	11	78	78	11	11	0	0	0	0
6	IVM	0	93	93	2	2	3	3	0	0	2	2
		14	11	11	72	72	17	17	0	0	0	0
	ALB	0	32	32	59	59	7	7	0	0	2	2
		14	30	30	45	45	25	25	0	0	0	0
7	IVM	0	28	28	11	11	13	13	0	0	48	48
		14	nd	nd	nd	nd	nd	nd	nd	nd	nd	nd
	ALB	0	37	37	29	29	12	12	0	0	22	22
		14	nd	nd	nd	nd	nd	nd	nd	nd	nd	nd
8	IVM	0	65	65	26	26	8	8	0	0	1	1
		14	nd	nd	nd	nd	nd	nd	nd	nd	nd	nd
	ALB	0	61	61	29	29	9	9	0	0	1	1
		14	nd	nd	nd	nd	nd	nd	nd	nd	nd	nd
9	IVM	0	83	83	10	10	5	5	0	0	2	2
		14	nd	nd	nd	nd	nd	nd	nd	nd	nd	nd
	ALB	0	81	81	10	10	6	6	0	0	3	3
		14	nd	nd	nd	nd	nd	nd	nd	nd	nd	nd
10	IVM	0	38	38	38	38	15	15	5	5	4	4
		14	nd	nd	nd	nd	nd	nd	nd	nd	nd	nd
	ALB	0	29	29	44	44	23	23	2	2	2	2
		14	nd	nd	nd	nd	nd	nd	nd	nd	nd	nd

*Abbreviation*: nd, no larvae were detected

### Egg hatch test

The hatching in the discriminating dose of 0.1 μg TBZ/ml were 89% and 74% in Farm 1 and Farm 2, respectively (Table 4). The EC_50_ value was 0.59 μg/ml for Farm 1 and 0.75 μg/ml for Farm 2.

**Table 4:** The results of the egg hatch test based on counting 100 eggs/larvae per well for each concentration of thiabendazole in Farm 1 and Farm 2

Thiabendazole concentration (μg/ml)	Farm 1		Farm 2	
	Average number of		Average number of	
	hatched eggs (larvae)	inhibited (dead) eggs	hatched eggs (larvae)	Inhibited (dead) eggs
0	94.0	6.0	95.5	4.5
0.01	84.0	16.0	78.5	21.5
0.025	88.0	12.0	76.5	23.5
0.05	87.5	12.5	75.5	24.5
0.1	89.0	21.0	74.0	26.0
0.2	77.0	23.0	70.0	30.0
0.3	63.5	36.5	62.5	37.5
0.5	49.5	50.5	44.5	55.5

## Discussion

Currently, control of GIN still relies heavily on regular treatments with anthelmintic drugs, but worm control is threatened by AR [6]. To enable sustainable parasite control in the future, it is important to use the available anthelmintics prudently. This study was undertaken to provide an evidence-based and a state-of-the-art picture of the AR situation for benzimidazoles and macrocyclic lactones in the Campania region (southern Italy) in ten dairy sheep farms chosen in the study area. Levamisole was not investigated in this study since no drugs are available in Italy containing only this molecule to control infections by GIN in sheep.

The distribution of egg counts between different animals of the same farm is well known to be overdispersed among hosts [29–31]. In particular, our study showed that a range from 1 to 600 EPG was seen in 70% of the sheep (Fig. 2). At animal level, our findings showed that a small number of sheep is responsible for shedding the majority of GIN eggs into the environment (range from 601 to 35,520 EPG). Such levels of overdispersion, likely due to multiple factors (e.g. based on parasite biology/epidemiology, farm management, treatment strategies, etc.), may provide an opportunity for using targeted selective treatments (TST) based on FEC [32].

On each farm, both classes of anthelmintics were tested on group sizes of 40 sheep/drug performing the FECRT according to pre- and post-treatment using the sensitive and accurate Mini-FLOTAC technique (detection limit = 5 EPG) on both individual and pooled samples. Previous studies [6, 14, 33, 34] have suggested that anthelmintic efficacy was high in southern Italy and supports the idea that with a correct management (the use of about two anthelmintic treatments in sheep per year) the development of resistance can be greatly reduced [14]. However, based on the FECRT results, AR was now detected in one and suspected in another farm (in Farm 1 efficacy of 75.0% with LL of 71.2% and in Farm 2 efficacy of 93.3% with LL of 93.0% were found) with the predominant GIN genus *Trichostrongylus* followed by *Haemonchus* at D14 for ALB. It should be noted that the results from the larval cultures may not accurately reflect actual worm burden or genera composition due to the influence of culture conditions. Therefore, molecular studies would be needed in future studies.

To confirm these results, the *in vitro* EHT was performed on GIN eggs from follow-up samples in Farm 1 and Farm 2 of the sheep treated with ALB. On both farms, the egg hatch inhibition at > 0.1 μg/ml TBZ was lower than 50% (Farm 1: 21%; Farm 2: 26%), which implies benzimidazole resistance [26, 35]. Additionally, to further confirm these findings, another *in vivo* trial for the FECRT on both suspicious farms was conducted two months after the first field trial with the similar outcome of reduced efficacy for administered fenbendazole with FECR values below 95% (Farm 1: 86.0%; Farm 2: 93.0%).

This approach of a follow-up trial using a different anthelmintic of the same class also excludes the phenomenon of present side resistance [36–40] and completes therefore the picture of present AR to the whole anthelmintic class. Finally, molecular detection of BZ-resistance associated polymorphisms in trichostrongyloids is a highly sensitive technique that allows to detect the manifestation of resistance alleles in the worm population and thus would be a further option to reassert resistance findings [26, 41].

The frequent, indiscriminate or inappropriate use of anthelmintic drugs to control GIN infection has resulted in selection of drug-resistant helminth populations. First, faecal examination should be done before anthelmintic treatment to assess the extent of parasite infection. FEC is rarely done in practice, which likely impacts many redundant anthelmintic treatments. Considering the treatment itself, underdosing is a common problem as weighing the sheep before deworming is far from being the routine practice. Dosage is usually calculated according to the weight estimated by farmers or veterinarians, which likely portrays the average weight of the flock rather than the weight of the heaviest animal. Therefore, underdosing is a great risk factor for the development of AR.

Furthermore, improper homogenization of the anthelmintic suspension and improper calibration of dosing equipment during treatment might result in underdosing. Due to the low treatment costs of benzimidazoles and the broad spectrum of action combatting also other helminths such as *F. hepatica*, *Dicrocoelium dendriticum*, lungworms and *Moniezia* spp., it is often used for several consecutive years. Comparing all tested farms, Farm 1 and Farm 2 having had a known history of fasciolosis relying therefore on the exclusive use of ALB, administered three to four times per year, between 2008 and 2014. High frequency of anthelmintic treatment has a major impact on speeding up the development of AR. While susceptible GIN are killed during treatment, species being resistant to the agent survive. With frequent deworming, using the same anthelmintic, resistant GIN have a selective advantage and make up an increasing proportion in the parasitic gene pool [42]. It can be assumed that the intensive use of ALB on Farm 1 and Farm 2 has contributed to the development of AR on both farms.

Moreover, the present study provides further insights into standardization of FEC and FECRT on pooled faecal samples in sheep. High correlation coefficients (Spearman) were found between the mean of individual samples and the mean of pooled samples when considering FEC at D0 (*r*_*s*_ = 0.984) and D14 (*r*_*s*_ = 0.913), whilst the Lin’s concordance correlation coefficients showed a substantial agreement at D0 (CCC = 0.973) and a poor agreement at D14 (CCC = 0.873) due a lot of zero data, since only two farms (Farm 1 and 2) showed AR. Despite this, high correlation and agreement coefficients (Spearman and Lin) were found between the mean EPG of individual samples and the mean EPG of pooled samples when considering overall FEC (D0 and D14) and FECR% according to [15, 17], which found that the pooling for both FEC and FECR test was a valid alternative of reducing labour and costs involved in both assessing infection intensity and diagnosing AR in sheep and cattle, respectively. Therefore, as there is evidence that the classifications of AR on pooled samples coincide with the results on individual samples, with particularly low variance when performed with Mini-FLOTAC, this study confirms this correlation and reasserts that pooled samples are cost-effective, time-saving alternatives [17, 18]. There is the need for a standardized procedure in order to be able to recognize resistance at an early stage. Since 1992, the FECRT has been the method of choice as an *in vivo* assay [6, 23, 35]. Its quality of performance is dependent on experimental setup, egg excretion at baseline, sample size and type of diagnostic tool but no definite recommendations can be made so far [18, 20, 43]. In this respect, the study unifies current recommendations and develops improvements towards a standardized protocol of AR detection.

When carrying out this study, care was taken to exclude common sources of error and avoided possible confounding factors unrelated to AR: the compilation of sheep for the treatment group happened randomly; high quality anthelmintics from renowned producers were used; correct dosage for each enrolled animal was assured through calculation based on individually weighing of the animals; and anthelmintics were administered carefully by experienced and trained veterinary practitioners following the instructions of the manufactures. For the FEC, the Mini-FLOTAC technique was used in this trial as it has been shown beneficial diagnostic performance combining sensitivity, accuracy and easy usage [19, 44].

Therefore, it is a convenient method for detecting the presence of low level of AR in ovine nematodes [18]. The evaluation of FECR with the *eggCounts-2.3* package in R version 3.6.1. showed a higher diagnostic performance and less susceptibility to error sources compared to the evaluation considering a control group [22]. Furthermore, the chosen sample size of 40 sheep per tested drug is large compared to the recommended sample size of *n* = 10–15 suggested by Coles et al. [23]. This larger sample size implies a high probability of sufficient baseline egg excretions, measured through an extrapolation of the model designed by Levecke et al. [20].

## Conclusions

The continuous monitoring of AR in sheep farms in southern Italy is essential, as anthelmintic efficacy is still high [14] and the development of AR needs to be detected early. In southern Italy the maintaining of *refugia* for susceptible nematode populations is the result of applying of methods for FECRT monitoring. Farmers should be properly informed about circumstances boosting the development of AR and receive appropriate training for responsible deworming management. The comparison of studies in Europe shows that the reflection of AR on farms is distorted by inadequate monitoring through biased choices of farms, insufficient number of tested drugs, or prevalent treatment failure [6]. Hence, the integration of a standardized, accurate and practical approach to monitor anthelmintic efficacy is required in order to compile a precise picture of the AR status worldwide. Likewise, further research is needed to develop alternative approaches to minimize the use of anthelmintics.

## Data Availability

All data generated or analysed during this study are included in this published article. The datasets used and/or analysed during the present study available from the corresponding author upon reasonable request.

## Abbreviations

ALB: albendazole
AR: anthelmintic resistance
ALB: albendazole
BZ: benzimidazoles
CCC: Lin’s concordance correlation coefficients
CI: confidence interval
COMBAR: COMBatting Anthelmintic Resistance in Ruminants
D0: pre-treatment
D14: post-treatment
DMSO: dimetyl sulfoxide
EC_50_: 50% egg hatch inhibition
EHT: egg hatch test, EPG: egg per gram of faeces
EPR: eprinomectin
FBZ: fenbendazole
FS: flotation solution
FEC: faecal egg count
FECRT: faecal egg count reduction test
GIN: gastrointestinal nematode
IVM: ivermectin
L3: third-stage larvae
LL: lower limit
ML: macrocyclic lactones
*r*_*s*_: Spearman’s rho correlation coefficient
TBZ: thiabendazole
TST: targeted selective treatment
TT: targeted treatment
